# Preparation of Novel Hierarchical Catalysts by Simultaneous Generation of β‐Zeolite and Mesoporous Silica for Catalytic Cracking

**DOI:** 10.1002/cplu.202400447

**Published:** 2024-10-29

**Authors:** Haruna Oshimura, Shuuma Tanaka, Shouya Nagata, Shinya Matsuura, Tadanori Hashimoto, Atsushi Ishihara

**Affiliations:** ^1^ Mie University 1577 Kurima Machiya-Cho Tsu City, Mie Prefecture 514-8507 Japan; ^2^ Mie Prefectural Industrial Research Institute 5-5-45 Takajyaya Tsu City, Mie Prefecture 514-0819 Japan

**Keywords:** Hierarchical catalyst, β-zeolite, Porous materials, Gel skeletal reinforcement, Catalytic cracking

## Abstract

The gel skeletal reinforcement (GSR) method was applied at the preparation stage of β‐zeolite to prepare a novel hierarchical catalyst. A solution of hexamethyldisiloxane (HMDS) and acetic anhydride, a GSR reagent, was added to the mixture of colloidal silica, sodium aluminate, tetraethylammonium hydroxide, sodium hydroxide and water, and successive aging and hydrothermal treatment gave microporous β‐zeolite surrounded by mesoporous silica like core‐shell structure. Its properties were characterized by XRD, nitrogen adsorption and desorption, NH_3_‐TPD, TEM, and TG‐DTA measurements, and further characteristics of the catalysts produced were clarified by the catalytic cracking of *n*‐dodecane. The hierarchical structure of microporous zeolite and mesoporous silica was shown from GSR‐2.9HS−H‐Beta to GSR‐3.2HS−H‐Beta, where the molar ratio of HMDS and silica source of β‐zeolite was 2.9~3.2 : 100. It was found that in the catalytic cracking of *n*‐dodecane, the relative activity (the conversion per the amount of zeolite crystals) increased with the increase in mesopore volume and surface area. The result indicated that the introduction of mesopores was effective even in catalytic cracking of small molecule of *n*‐dodecane.

## Graphical abstract

Simultaneous generation of β‐zeolite and mesoporous silica using a solution of hexamethyldisiloxane (HMDS, HS) and acetic anhydride (AA), a gel skeletal reinforcing (GSR) agent, gave a novel hierarchical catalyst where microporous β‐zeolite was surrounded by mesoporous silica like core‐shell structure. Relative activity in catalytic cracking increased with developing the mesoporous structure.

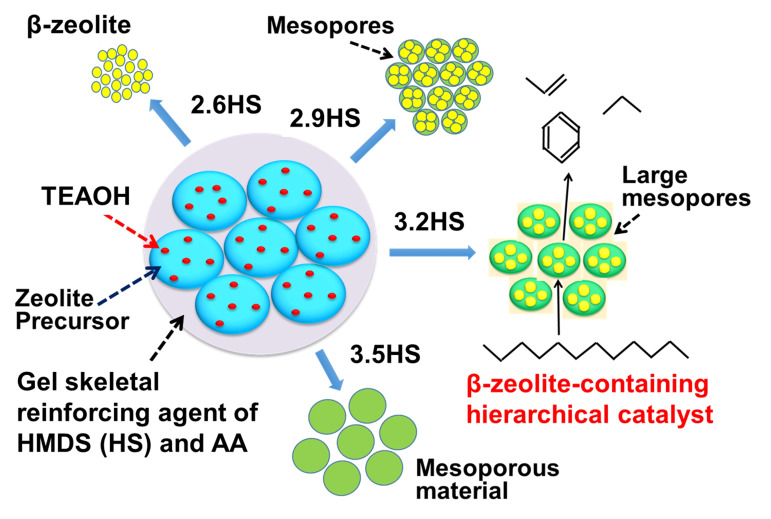

## Introduction

Recently many researches have reported catalytic cracking of heavier fractions.^[1]^ However, most of examples treated zeolite itself rather than a matrix or a whole catalyst. Further, ZSM‐5‐[^2^, ^3^, ^4^, ^5^, ^6^, ^7^, ^8^, ^9^, ^10^, ^11^, ^12^, ^13^, ^14^, ^15^, ^16^, ^17^, ^18^, ^19^, ^20^] and Y‐zeolites[^21^, ^22^, ^23^, ^24^, ^25^, ^26^, ^27^, ^28^, ^29^, ^30^, ^31^, ^32^, ^33^, ^34^, ^35^, ^36^] are the main component of catalysts and the preparation of their hierarchical ones have been major subjects. Therefore, examples using other zeolites like β‐zeolite are very few.[^37^, ^38^, ^39^, ^40^, ^41^, ^42^, ^43^] Further, although industrial FCC catalysts consisted of not only zeolite but also matrix and other components,^[44]^ examples investigating matrix itself[^45^, ^46^, ^47^, ^48^, ^49^, ^50^, ^51^, ^52^, ^53^, ^54^, ^55^, ^56^] are very few.

Matrices used in FCC catalysts are required to have various features such as wearing resistance, fluidization properties, high‐temperature hydrothermal stability, metal resistance,^[57]^ and an optimal pore structure for diffusion of reactants and products. Among them, amorphous matrices are attracting attention because they can more selectively decompose heavy oil molecules that cannot be fully decomposed by zeolite pores in catalytic cracking.^[1]^ An amorphous oxide such as silica, silica‐alumina etc. is one of the common active matrices in FCC, and could be produced by the sol‐gel method.^[58]^ Such oxide has the advantage that its activity and selectivity can be controlled by changing properties such as pore structure.^[1]^ In order to fabricate a new type of matrix with larger pores, we have developed a gel skeletal reinforcement (GSR) method,^[59]^ which can produce a highly large porous material without using a supercritical fluid. The GSR method is a method that can reinforce the skeletal structure of silica‐gel and minimize the pore shrinkage by aging the gel with a reinforcing agent before drying. The aging process strengthens the network of gels to provide strength that can withstand stress, and the dissolution and reprecipitation of silica particles (Ostwald growth) causes particle growth. Furthermore, the condensation is suppressed by reinforcing the OH group on the gel surface with a trialkyl‐ or a trialkoxy‐silyl group. These phenomena are thought to lead to the formation of large particles and large pores, and the GSR method is expected to be a method for creating new porous materials.

In our research, we have reported that amorphous silica and silica‐alumina with large pores can be produced by applying various skeletal reinforcements at the gel preparation stage using the sol‐gel method.[^1^, ^12^, ^14^, ^16^, ^22^, ^59^] Furthermore, by introducing a GSR method at the production stage of ZSM‐5 zeolite, a zeolite‐containing hierarchical catalyst where microporous zeolite was surrounded by mesoporous silica like a core‐shell structure was produced, and excellent activity and selectivity were obtained in the catalytic cracking of *n*‐dodecane and LDPE.[^12^, ^16^]

In the present study, we tried to synthesize β‐zeolite and amorphous silica simultaneously in the presence of GSR reagent of a hexamethyldisiloxane (HMDS) and acetic anhydride (AA) solution in one step under the hydrothermal synthesis condition using tetraethylammonium hydroxide (TEAOH), and investigated the change in its activity and selectivity by changing the pore structure from conventional zeolite. A novel β‐zeolite‐containing hierarchical catalyst where microporous β‐zeolite was surrounded by mesoporous silica could successfully be produced and exhibited the good performance in catalytic cracking of *n*‐dodecane.

## Results and Discussion

### Characterization of β‐Zeolite and β‐Zeolite‐Containing Hierarchical Catalysts Using Gel Skeletal Reinforcement

**Figure 1:**
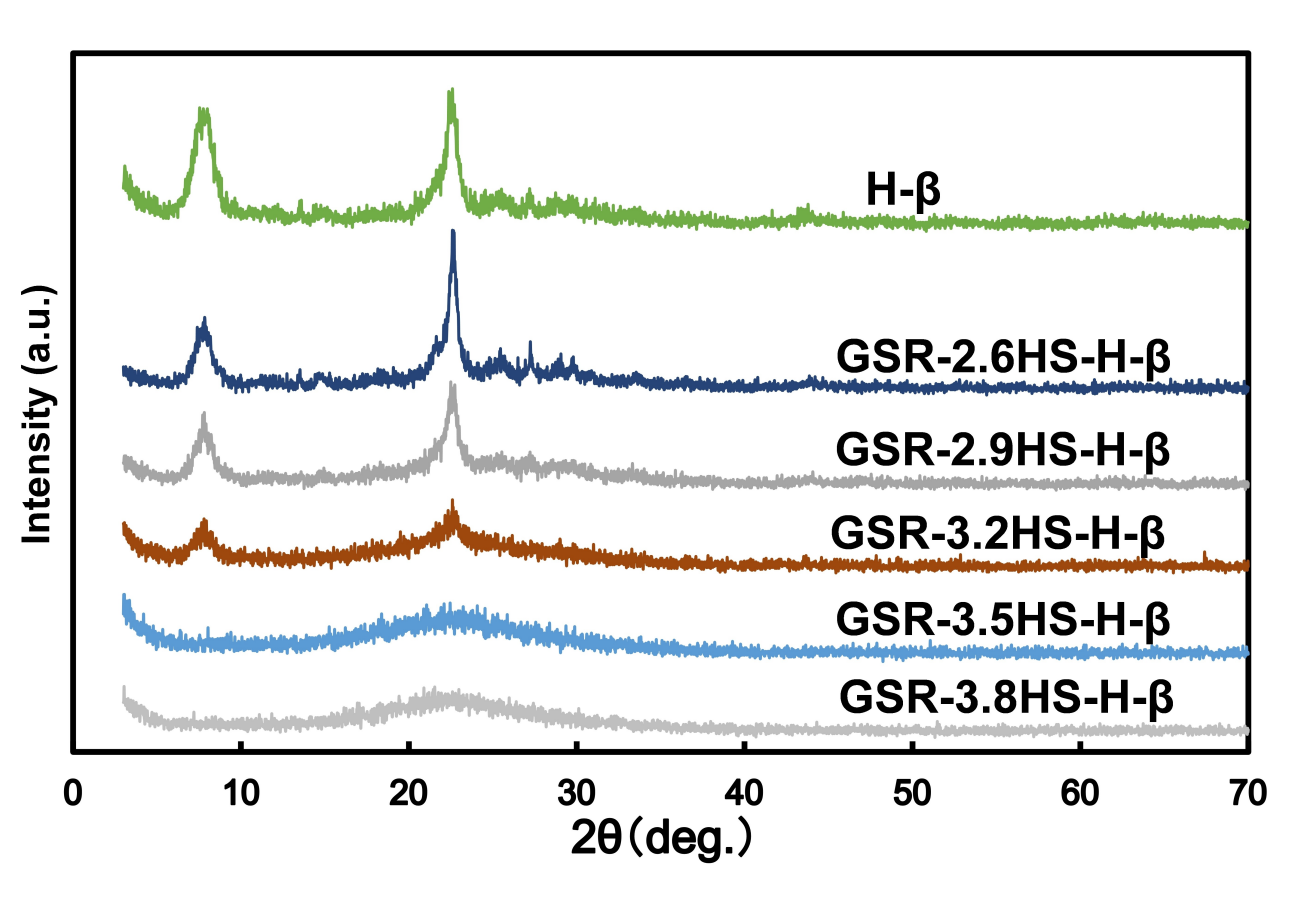
XRD patterns of β‐zeolite and β‐zeolite‐containing hierarchical catalysts.

Figure 1 and Figure S1 show the results from XRD measurement of catalysts after and before cation exchange, respectively. As shown in Figure 1, the peak intensity of GSR‐2.6HS−H‐β was confirmed to be the same as that of H‐Beta without gel skeletal reinforcement. In GSR‐2.9HS−H‐β, the peak intensity was reduced compared to GSR‐2.6HS−H‐β. In GSR‐3.2HS−H‐β, the peak intensity was significantly reduced, and a slight peak was observed. No peaks were observed in GSR‐3.5HS−H‐β and GSR‐3.8HS−H‐β, indicating that these catalysts would be in the amorphous phase. Compared with the results for catalysts without ion exchange in Figure S1, no reduction in peaks due to ion exchange was observed.

Table 1 shows the results of XRF measurement. In all samples, Na_2_O was no longer seen after cation exchange, indicating that all Na^+^ was removed as a result of cation exchange. Table 1 also summarizes the SiO_2_/Al_2_O_3_ ratio obtained from XRF measurement. The SiO_2_/Al_2_O_3_ ratios were in the range 32–36 near to original ratio of 32. No change in SiO_2_/Al_2_O_3_ ratio due to changes in the concentration of the gel reinforcing agents was confirmed.

**Table 1:**
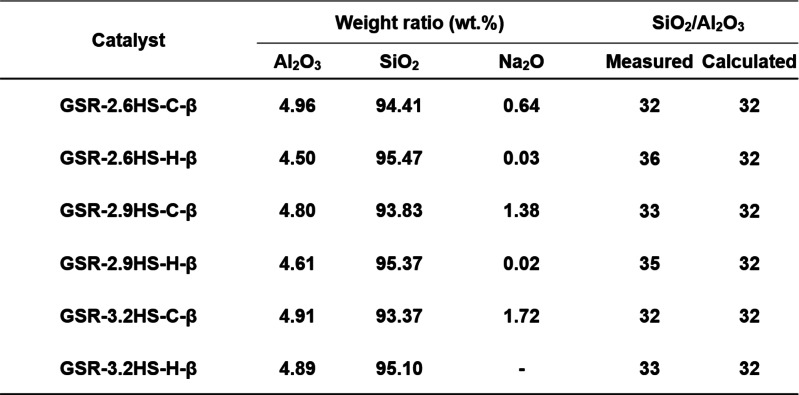
XRF measurement of SiO_2_/Al_2_O_3_ for β‐zeolite‐containing hierarchical catalysts.

**Figure 2:**
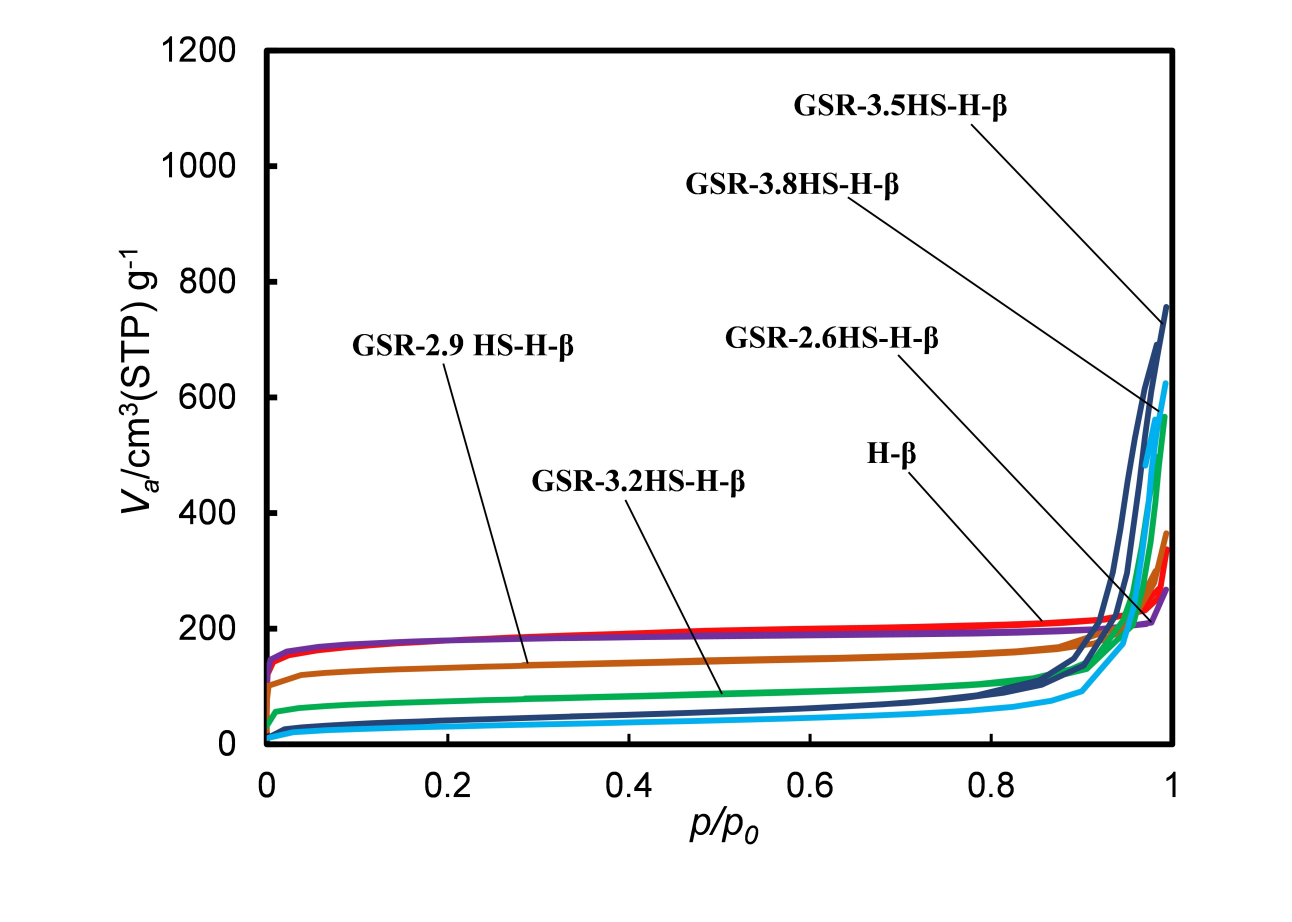
Nitrogen adsorption‐desorption isotherms of β‐zeolite and β‐zeolite‐containing hierarchical catalysts.

**Figure 3:**
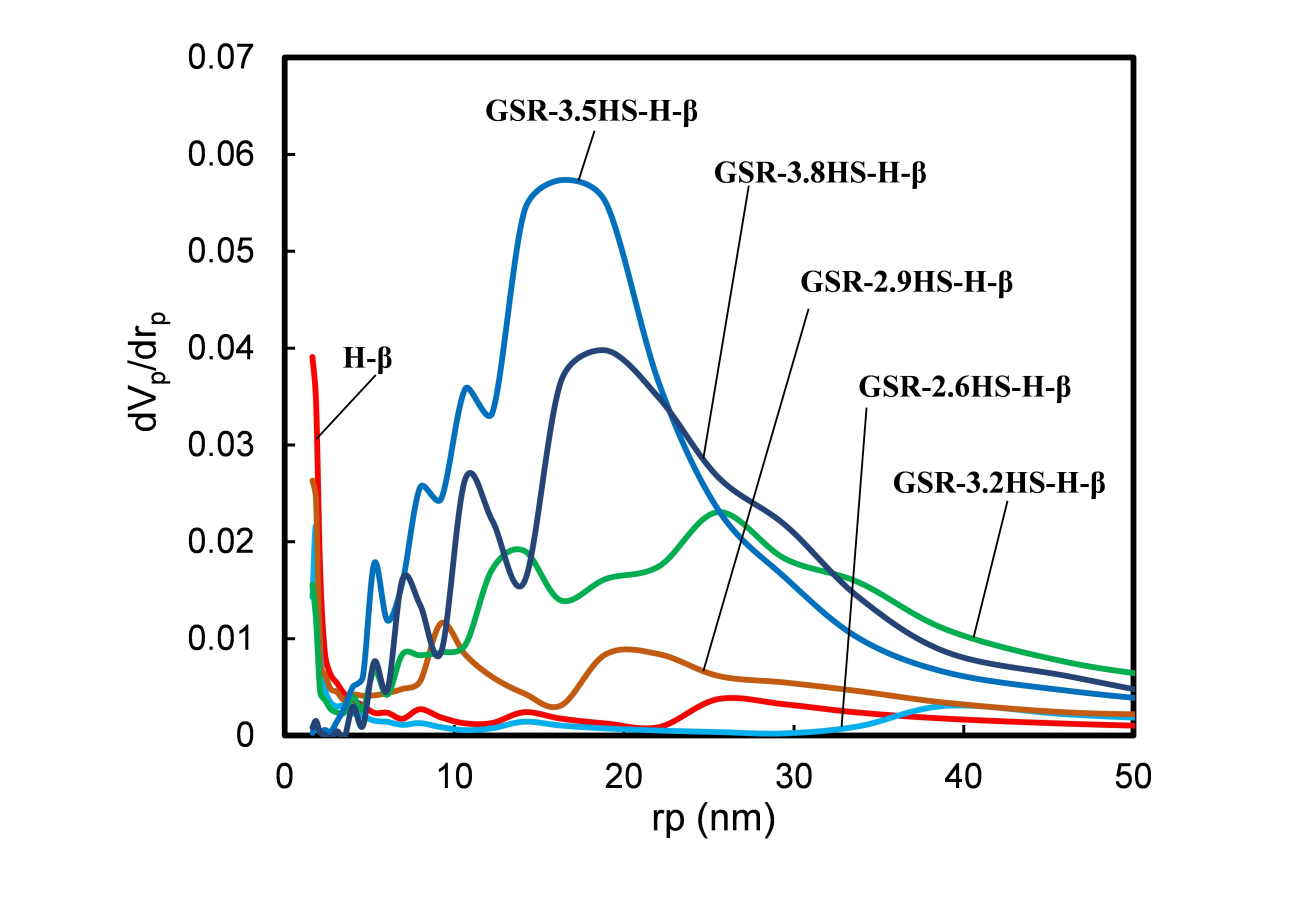
BJH pore‐size distribution of β‐zeolite and β‐zeolite‐containing hierarchical catalysts.

Table 2, Figures 2, 3, Figure S2 and Figure S3 show results from nitrogen adsorption and desorption measurement of catalysts. Figure 2 and Figure S2 show the adsorption and desorption isotherms of the prepared H‐type and C‐type samples, respectively. It can be seen that GSR‐2.6HS−H‐β had adsorption to the same extent as H‐β without gel skeletal reinforcement at a relative pressure of around 0, and most of the adsorption are derived from micropores. In GSR‐2.9HS−H‐β, the amount of adsorption slightly decreased at a relative pressure of around 0 and the amount of adsorption increased at a relative pressure of 0.8–1, suggesting that it would be derived from openings between zeolite crystals. In GSR‐3.2HS−H‐β, GSR‐3.5HS−H‐β, and GSR‐3.8HS−H‐β, the amount of adsorption at a relative pressure of around 0 was further reduced, and the amount of adsorption at a relative pressure of 0.8–1 increased. GSR‐3.2HS−H‐β still had micropores which were shown as crystals in XRD of Figure 1. However, GSR‐3.5HS−H‐β and GSR‐3.8HS−H‐β no longer exhibited adsorption at around 0, indicating that micropores would not grow as shown in XRD of Figure 1. As shown in Figure S2, almost similar results in the nitrogen adsorption and desorption isotherms were obtained for catalysts of GSR−C‐β. However, GSR‐3.2HS−C‐β, GSR‐3.5HS−C‐β and GSR‐3.8HS−C‐β before cation exchange exhibited slight larger adsorptions around a relative pressure of around 0 compared with GSR‐3.2HS−H‐β, GSR‐3.5HS−H‐β and GSR‐3.8HS−H‐β after cation exchange, indicating that microporous SiO_2_ particles would have been present in the formers and that during the ion‐exchange operation, those could have been lost. These results could also be confirmed by values in Tables 2 and S1.

**Table 2:**
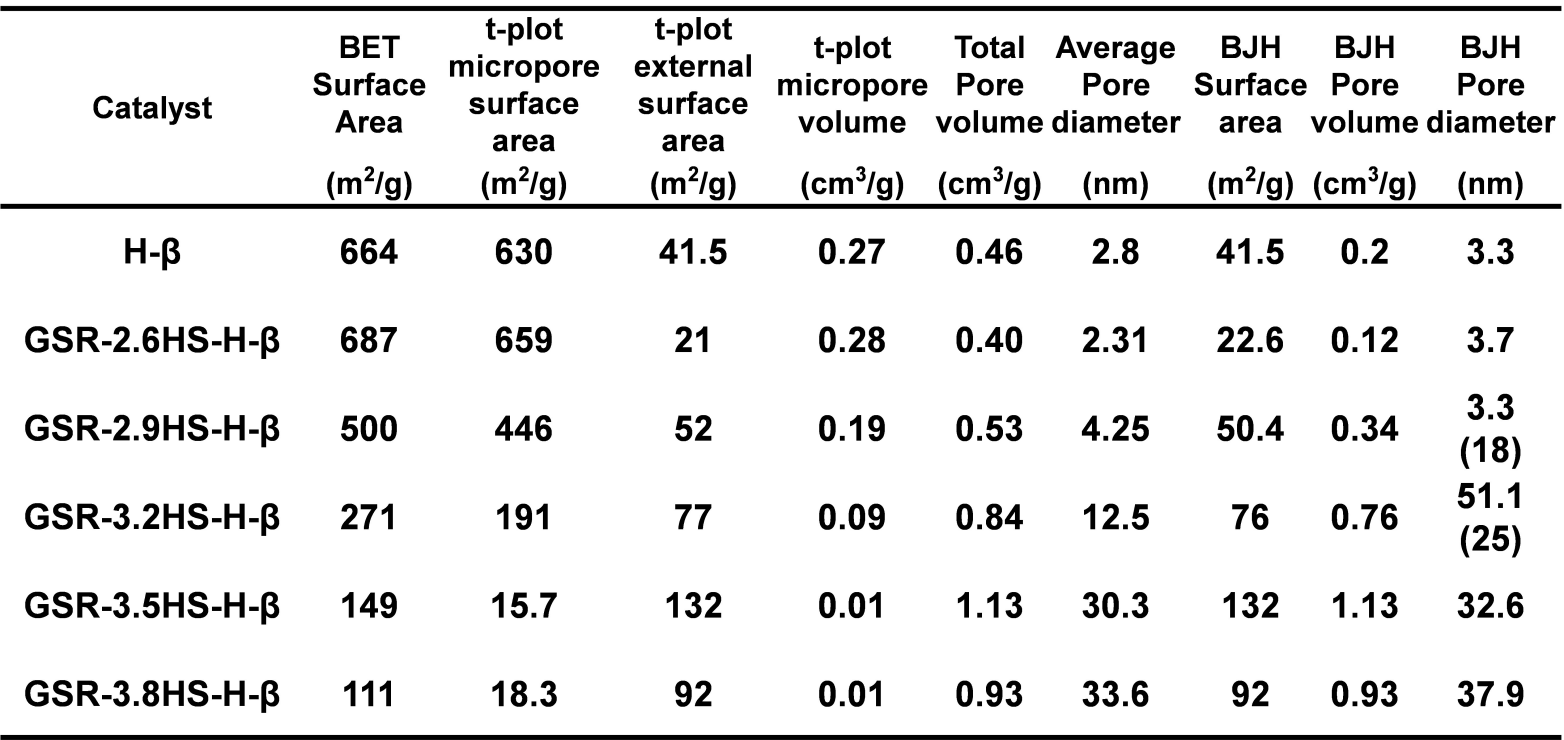
Pore properties of β‐zeolite and β‐zeolite‐containing hierarchical catalysts obtained by nitrogen adsorption‐desorption measurement.

Figures 3 and S3 shows the BJH pore size distributions of the prepared H‐type and C‐type samples, respectively. H‐β, GSR‐2.6HS−H‐β, GSR‐2.9HS−H‐β and GSR‐3.2HS−H‐β as well as C‐β, GSR‐2.6HS−C‐β, GSR‐2.9HS−C‐β and GSR‐3.2HS−C‐β exhibited the distribution of mesopores in the range 3–4 nm near intercept, which would be derived from openings of β‐zeolite crystals because these samples included β‐zeolite. Large mesopores developed in the order GSR‐2.9HS−H‐β, GSR‐3.2HS−H‐β and GSR‐3.5HS−H‐β while large mesopores of GSR‐3.8HS−H‐β decreased. Similar results were obtained for C‐type samples, indicating that these large mesopores were maintained during the ion exchange operation.

Tables 2 and S1 show the pore characteristics of various samples including BET surface area, micropore surface area by t‐plot, external surface area by t‐plot, micropore volume by t‐plot, total pore volume, average pore diameter, BJH surface area, BJH pore volume and BJH pore diameter. GSR‐2.6HS−H‐β was found to be composed of mostly micropores of β‐zeolite and was very similar to H‐β. In GSR‐2.9HS−H‐β, the mesopores increased, and a second peak of mesopore was observed at 18 nm, suggesting that a hierarchical structure was formed as described above. In GSR‐3.2HS−H‐β, GSR‐3.5HS−H‐β and GSR‐3.8HS−H‐β, broad peaks were observed in the range 30–50 nm, suggesting that not only mesopores but also macropores developed. As the amounts of reinforcing agents increased, the micropores decreased and the mesopores increased. Total pore volume, BJH pore volume and the average pore diameter also increased with an increase in the amounts of reinforcing agents. As shown in Table S1, GSR−C‐β samples exhibited similar results to those of GSR−H‐β. On the other hand, micropores in GSR‐3.2HS−C‐β, GSR‐3.5HS−C‐β, and GSR‐3.8HS−C‐β were lost after the ion exchange while the mesopores were almost unchanged, suggesting that most of micropores in these catalysts would have consisted of amorphous phase. Since GSR‐3.2HS−C‐β had β‐zeolite crystals, however, the decrease in the micropores was inhibited.

**Figure 4:**
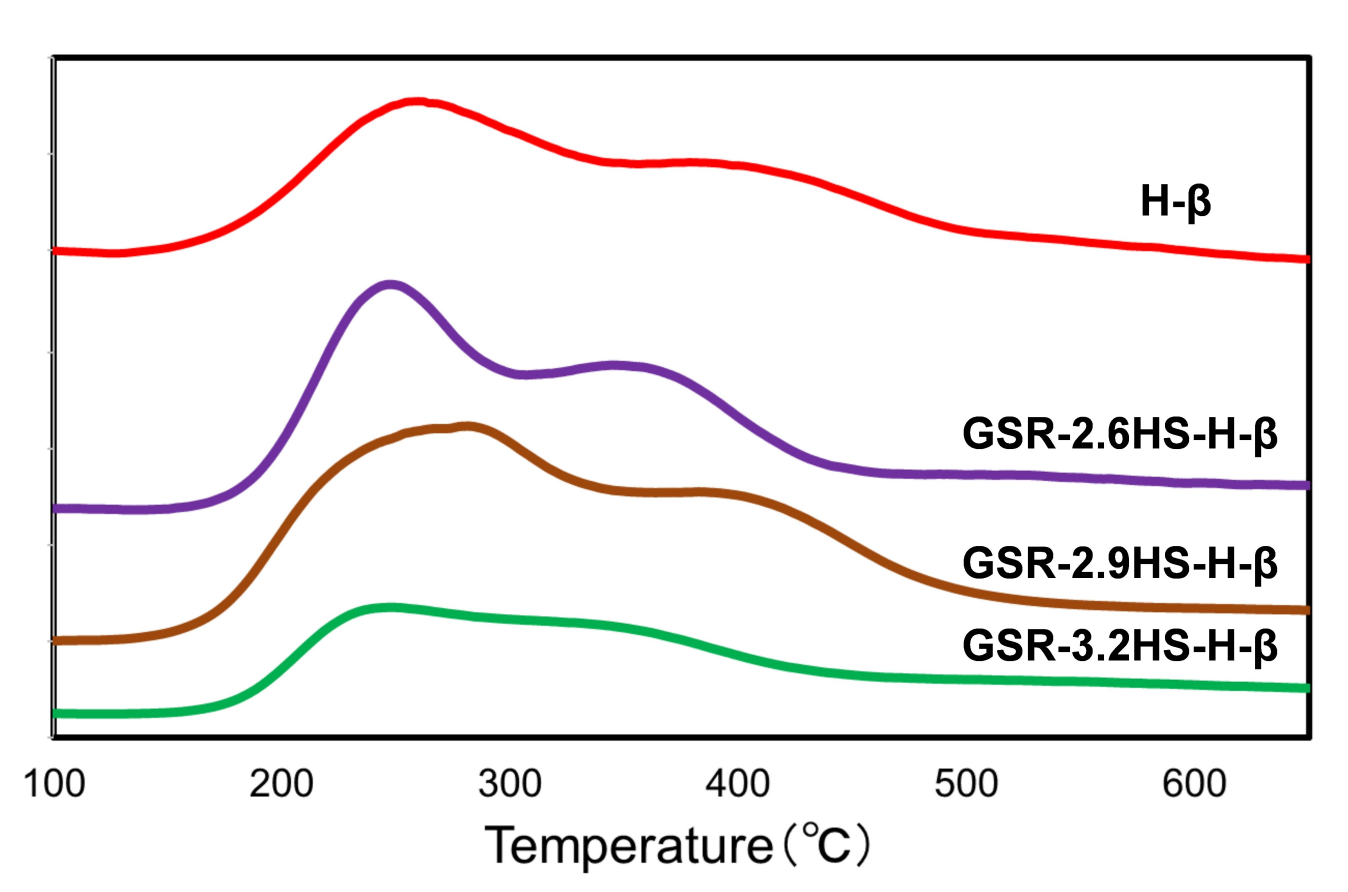
NH_3_‐TPD patterns of β‐zeolite and β‐zeolite‐containing hierarchical catalysts.

Figure 4 and Table 3 show results from NH_3_‐TPD measurement. Total amounts of NH_3_ desorbed were lower than amounts of Al calculated from XRF data in Table 1. The amounts of NH_3_ desorbed for GSR‐2.6HS−H‐β and GSR‐2.9HS−H‐β were near to each amount of Al. As the presence of Al forms the acid site in zeolite, the amount of acid sites should be very near or same as the amount of Al in the highly dispersed state of Al in zeolite crystals. Total amount of NH_3_ desorbed for GSR‐3.2HS−H‐β was significantly lower than the amount of Al calculated from XRF data, indicating that as the reinforcing agents were larger, Al atoms would be isolated as Al_2_O_3_ in mesoporous SiO_2_., which could lead to the decrease in amounts of NH_3_ desorbed.

**Table 3:**
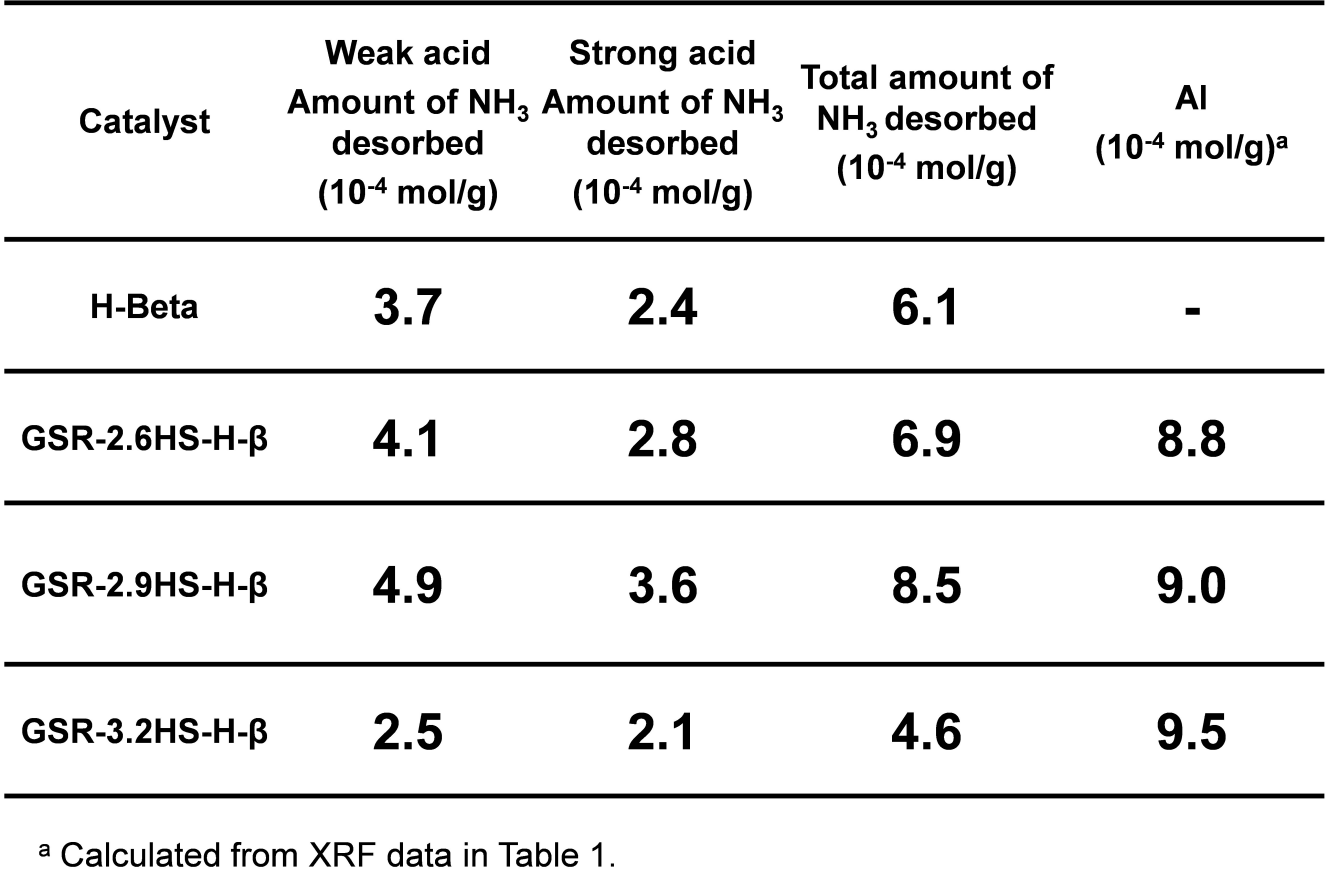
Amounts of NH_3_ desorbed of β‐zeolite and β‐zeolite‐containing hierarchical catalysts.

As two peaks are observed in NH_3_‐TPD, the amounts of NH_3_ desorbed at the lower and higher temperature can be regarded as weak acid sites and strong acid sites.^[50]^ It is generally thought that strong acid sites would be effective for catalytic cracking. Therefore, when strong acid sites increase the activity increases. The amount of strong acid sites for GSR‐3.2HS−H‐β was also lower than those for GSR‐2.6HS−H‐β and GSR‐2.9HS−H‐β, suggesting that the conversion of GSR‐3.2HS−H‐β could be lower than those of GSR‐2.6HS−H‐β and GSR‐2.9HS−H‐β. As shown below, the conversion in catalytic cracking for GSR‐3.2HS−H‐β decreased in comparison with those of GSR‐2.6HS−H‐β and GSR‐2.9HS−H‐β. Although NH_3_‐TPD does not distinguish Brønsted acid site and Lewis acid site, it is thought that both Brønsted and Lewis acid sites could be related to the activity of catalytic cracking because both acid sites could exchange each other in the presence of water at as high temperature as 500 °C.^[44]^

**Figure 5:**
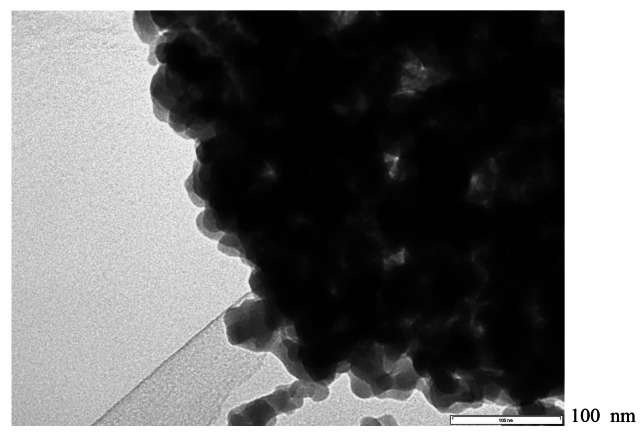
TEM image of GSR‐3.2HS−H‐β.

Figure 5 shows the TEM image of GSR‐3.2HS−H‐β. The highly permeable parts of the TEM images were derived from the porous structure of silica, and the poorly permeable parts were derived from the dense structure of β‐zeolite crystals with relatively uniform particles of 10–20 nm. GSR‐2.6HS−H‐β hardly had a highly permeable part and showed dense β‐zeolite crystals. GSR‐2.9HS−H‐β showed both dense β‐zeolite crystals and fine particles of β‐zeolite surrounded by amorphous SiO_2_. Only GSR‐3.2HS−H‐β showed fine particles of β‐zeolite surrounded by amorphous SiO_2_ as a major part of the image.

When compared with corresponding GSR‐xHS‐ZSM‐5 series catalysts reported previously,[^12^, ^16^] GSR‐10HS‐ZSM‐5 was composed of mostly ZSM‐5 crystals. GSR‐12HS‐ZSM‐5 slightly grew mesoporous SiO_2_ with significant amount of ZSM‐5 while GSR‐13HS‐ZSM‐5 slightly grew ZSM‐5 with significant amount of mesoporous SiO_2_. Further, GSR‐14HS‐ZSM‐5 no longer grew ZSM‐5 crystals but grew only large mesoporous SiO_2_. Compared with the formation of ZSM‐5 in GSR‐xHS‐ZSM‐5, the formation of β‐zeolite in GSR‐xHS‐β was inhibited by the smaller amount of HMDS and AA added, suggesting that crystallization of β‐zeolite would somewhat be difficult to occur although temperature in preparation was slightly lower for GSR‐xHS‐β. SiO_2_/Al_2_O_3_ ratio was also lower for GSR‐xHS‐β, which may lead to the inhibition of crystallization of zeolite. It is known that when the amount of Al component was larger, the inhibition of the crystallization often occurred.^[60]^

### Catalytic Cracking Reaction of *n*‐Dodecane Using β‐Zeolite and β‐Zeolite‐Containing Hierarchical Catalysts Using Gel Skeletal Reinforcement

**Figure 6:**
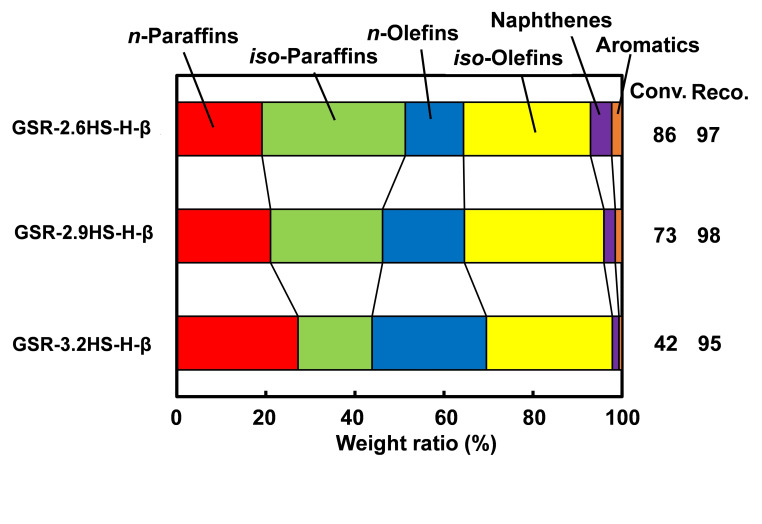
Selectivity for Paraffins, Olefins, Naphthenes and Aromatics on catalytic cracking of *n*‐dodecane using β‐zeolite‐containing hierarchical catalysts.

Figure 6 shows the selectivity for paraffins, olefins, naphthenes and aromatics on catalytic cracking of *n*‐dodecane using β‐zeolite containing hierarchical catalysts. As the amounts of reinforcing agents increased, the selectivity for paraffins, naphthenes and aromatics decreased and the selectivity for olefins increased. These results indicated that both hydrogen transfer and cyclization would be suppressed in GSR‐3.2HS−H‐β more significantly compared to GSR‐2.6HS−H‐β because of the decrease in the acid density of the catalyst as shown in the data of NH_3_‐TPD mentioned above. Similar observation was reported for GSR‐xHS‐ZSM‐5 series catalysts where ZSM‐5 and mesoporous silica were generated simultaneously in the presence of reinforcing agents of HMDS and AA and tetrapropylammonium hydroxide (TPAOH).[^12^, ^16^] These results indicated that the acid sites on the external surface of zeolite crystals would be effectively covered by the reinforcing agents and that the growth of zeolite crystals would be inhibited in the presence of reinforcing agents, which could suppress the hydrogen transfer and increase the selectivity for olefins.

**Figure 7:**
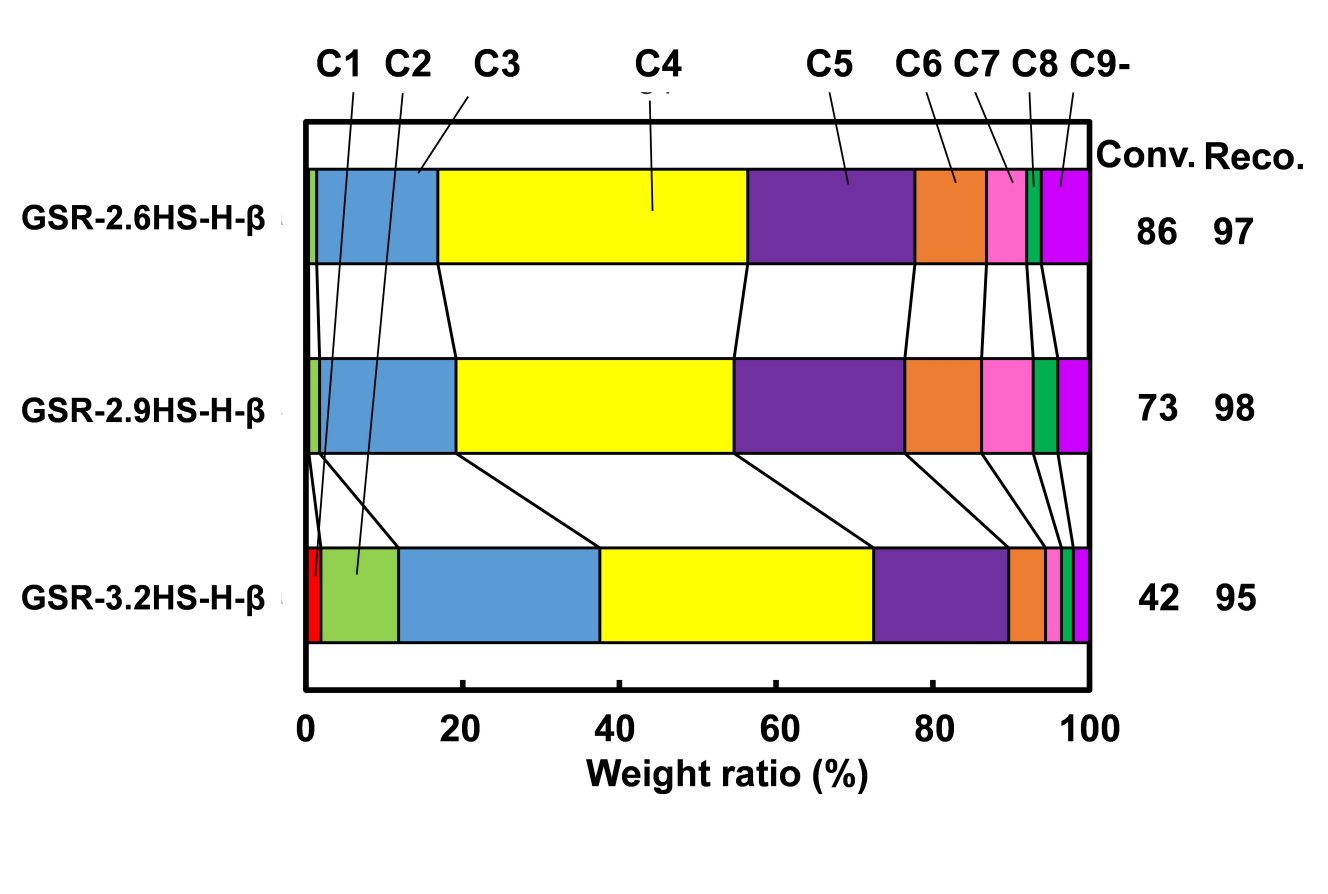
Distribution of carbon numbers on catalytic cracking of *n*‐dodecane using β‐zeolite‐containing hierarchical catalysts.

Figure 7 shows the distribution of carbon numbers on catalytic cracking of *n*‐dodecane using β‐zeolite containing hierarchical catalysts. There was little change in the carbon number distribution between GSR‐2.6HS−H‐β and GSR‐2.9HS−H‐β. In contrast, the gas products of C1‐C4 increased and the gasoline fraction decreased in GSR‐3.2HS−H‐β. Similar tendency in the distribution of carbon numbers was also observed when x for GSR‐xHS‐ZSM‐5 increased.[^12^, ^16^] GSR‐2.6HS−H‐β almost consisted of only β‐zeolite and its carbon number distribution as well as conversion in catalytic cracking of *n*‐dodecane were very similar to those for commercial β‐zeolite with similar SiO_2_/Al_2_O_3_ ^[52]^ except for olefin selectivity.

Table 4 summarizes the product distribution and catalytic properties of β‐zeolite containing hierarchical catalysts in catalytic cracking of *n*‐dodecane. The gasoline fraction decreased as the amounts of reinforcing agents increased, indicating that the ability of cracking on an active site would have increased. This was confirmed when the relative activity was estimated on the basis of the following equation:

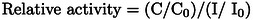




**Table 4:**
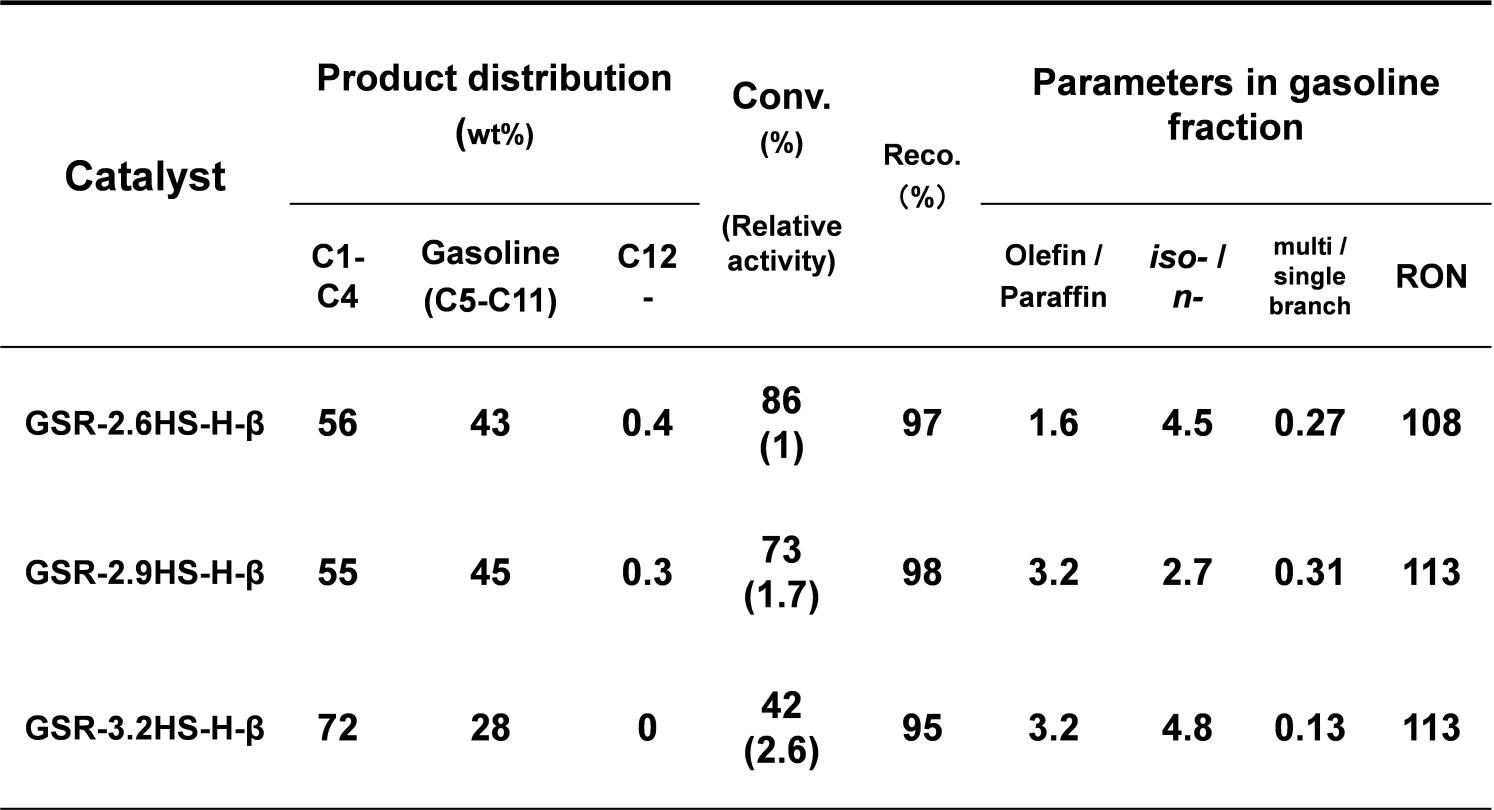
Product distribution and catalytic properties of β‐zeolite‐containing hierarchical catalysts in catalytic cracking of *n*‐dodecane.

where C=Conversion of a catalyst; C_0_=Conversion of GRS‐2.6HS−H‐β; I=Integral intensity of a catalyst for a XRD signal at 2θ of 23°; I_0_=Integral intensity of GRS‐2.6HS−H‐β for a XRD signal at 2θ of 23°.

The conversion decreased with increasing the amounts of reinforcing agents and was a maximum of 86 % for GSR‐2.6HS−H‐β. However, the relative activity (the numerical value in parenthesis in Table 4) calculated by above equation increased with increasing the amounts of reinforcing agents. It can be seen that the relative activity for one active site of zeolite increased with the increase in the amounts of reinforcing agents, and the introduction of mesopores efficiently carried out the introduction of the feed and the diffusion of products or intermediate molecules, which promoted the secondary cracking in the other active site of zeolite. In addition, the olefin/paraffin ratio in the gasoline fraction increased with an increase in the amount of reinforcing agents, indicating that the isolation of active site by mesoporous SiO_2_ generated by the reinforcing agents would inhibit the hydrogen transfer and promote the rapid diffusion of the active substance like olefins. In addition, relatively high RON values were obtained for GSR‐2.9HS−H‐β and GSR‐3.2HS−H‐β and further the high conversion was maintained for GSR‐2.9HS−H‐β.

Table 5 tabulates the coke yield of β‐zeolite containing hierarchical catalysts measured by TGA after catalytic cracking of *n*‐dodecane. The coke yield decreased in the order GSR‐2.6HS−H‐β, GSR‐2.9HS−H‐β and GSR‐3.2HS−H‐β, indicating that the catalyst with the higher relative activity could inhibit the coke formation.

**Table 5:**
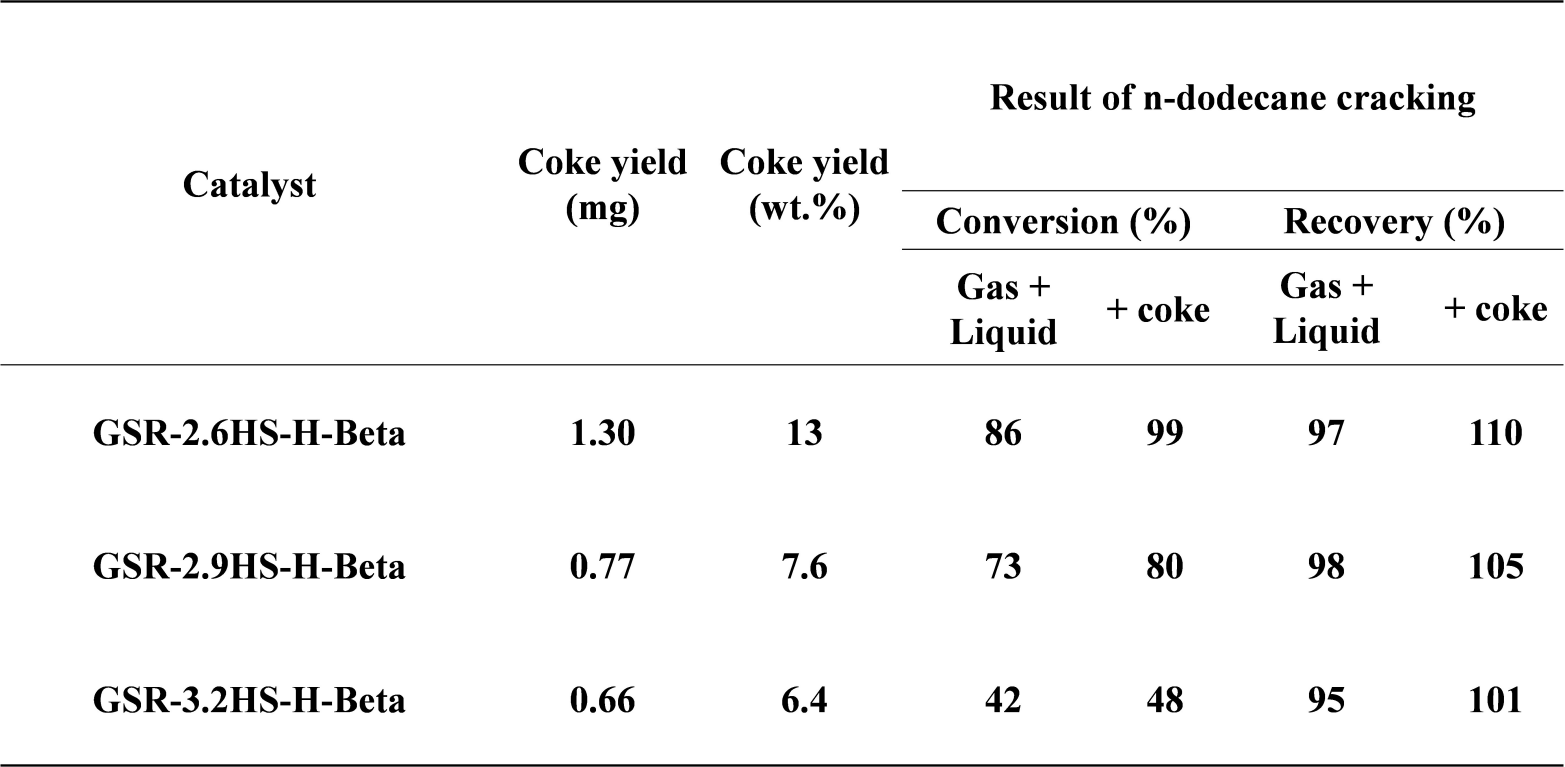
Coke yield of β‐zeolite‐containing hierarchical catalysts measured by TGA.

Cat.:10 mg, Temp.: 400–600 °C, Atmosphere: O_2_, Programming rate: 10.0 °C/min, Retention time at 600 C: 0 h.

**Figure 8:**
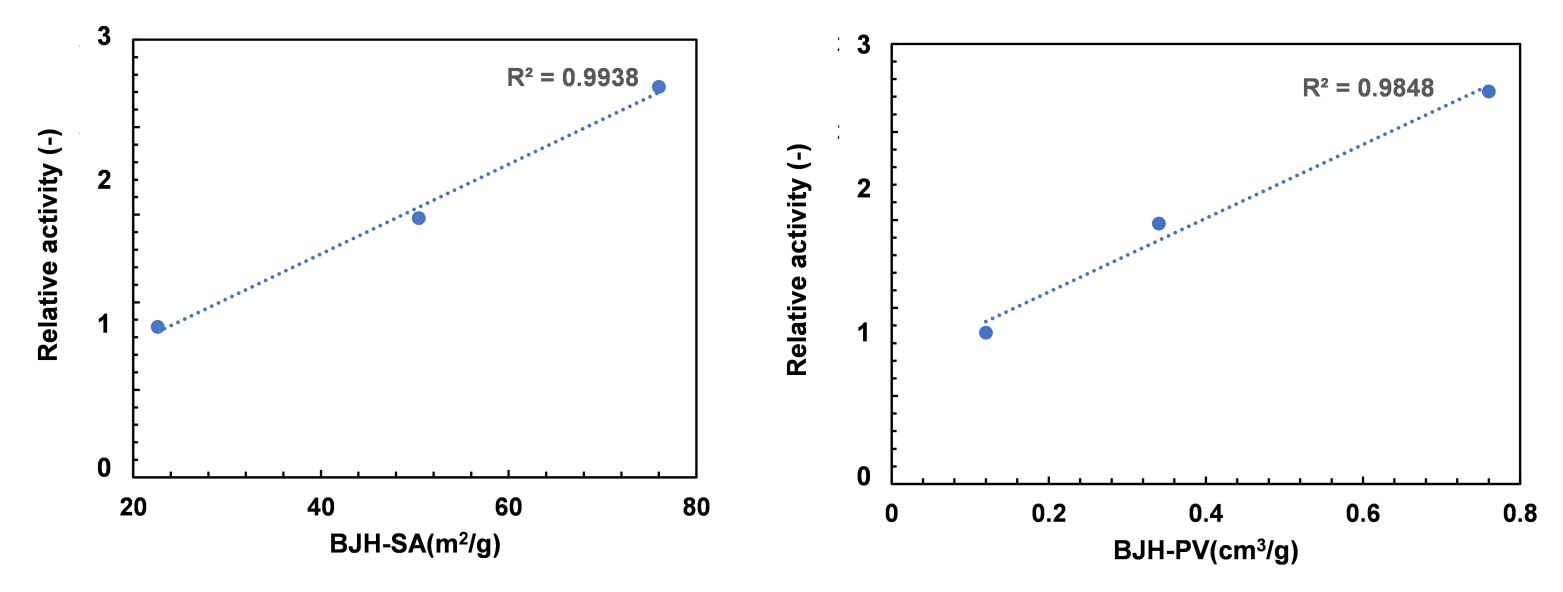
Correlation diagram between relative activity and pore structure.

Figure 8 shows the correlation between the relative activity and BJH‐surface area or BJH‐pore volume of each sample. It can be seen that the relative activity increased in proportion to the increase in BJH‐surface area and BJH‐pore volume, which represent the amount of mesopores. The result indicated that the higher relative activity could strongly be related to the size of mesopores in the catalyst. The results from the relationship between relative activity vs. mesoporous structure in GSR‐xHS‐β catalysts were consistent with those of corresponding GSR‐xHS‐ZSM‐5 catalysts.[^12^, ^16^]

There are very few examples treating with β‐zeolite‐containing hierarchical catalysts in catalytic cracking[^61^, ^62^, ^63^, ^64^, ^65^, ^66^, ^67^, ^68^, ^69^, ^70^, ^71^, ^72^, ^73^, ^74^, ^75^, ^76^, ^77^] although there are plenty of reports for Y and ZSM‐5. Further, many researchers have used hierarchical β‐zeolites, which basically consisted of only β‐zeolite, for more than 15 years while we used β‐zeolite‐containing hierarchical catalysts where not only β‐zeolite but also mesoporous silica were included. These reports showed the enhancement in the catalytic activity for not only polymer, bulky molecules, light cycle oil[^61^, ^62^, ^66^, ^67^, ^69^, ^70^, ^72^, ^73^] but also small molecules.[^63^, ^64^, ^65^, ^68^, ^74^] Fabrication of hierarchical β‐zeolites using a surfactant, a silanization agent[^61^, ^62^, ^70^, ^72^] and desilication^[66]^ and their superiority to polymers cracking have been reported. Metal supported hierarchical β‐zeolites were also used for dehydrogenation or cracking of *n*‐hepatene and *n*‐dodecane.[^63^, ^64^, ^65^] Commercial potential of hierarchical zeolites has also been discussed^[77]^ and hierarchical catalysts with β‐zeolites, which have larger micropores than ZSM‐5 and smaller micropores than Y‐zeolite, could have become one of candidates for commercial use while the development has just begun.[^1^, ^38^, ^67^, ^72^, ^75^, ^76^] Further development of hierarchical catalysts using β‐zeolites has been expected in catalytic cracking.

## Conclusions

In this study, we prepared novel β‐zeolite‐containing hierarchical catalysts, where a β‐zeolite component with micropores was surrounded by a matrix component of mesoporous silica like core‐shell structure, and evaluated the catalytic cracking of *n*‐dodecane. From the results of XRD and N_2_ adsorption and desorption measurements, it was found that GSR‐2.9HS−H‐β and GSR‐3.2HS−H‐β showed the presence of both β‐zeolite and mesoporous silica, and that a hierarchical pore structure developed. In GSR‐3.2HS−H‐β, the peak position in BJH mesopore size distribution was 50 nm, indicating that not only mesopores but also macropores developed. In the catalytic cracking of *n*‐dodecane, the conversion increased in the order GSR‐3.2HS−H‐β<GSR‐2.9HS−H‐β<GSR‐2.6HS−H‐β. With the increase in mesopores, however, the relative activity (the activity per zeolite crystal) increased and reached 2.6 for GSR‐3.2HS−H‐β while 1 for GSR‐2.6HS−H‐β, indicating that the introduction of mesopores could promote the diffusion of not only feed molecule but also reactive products and further secondary cracking on dispersed zeolite.

## Experimental Section

### Materials in Fabrication of β‐Zeolite and β‐Zeolite‐Containing Hierarchical Catalysts

Materials in fabrication of β‐zeolite and related hierarchical catalysts were 30.5 % colloidal silica (SiO_2_; Manufactured by JGC Catalyst Chemical Co., Ltd.), 65 % sodium aluminate (NaAlO_2_; Manufactured by Nacalai Tesque Lnc.), 35 % tetraethylammonium hydroxide (C_8_H_21_NO) as a structural determinant (hereinafter referred to as TEAOH; Manufactured by Kanto Chemical Co., Ltd.), sodium hydroxide (NaOH; Manufactured by Nacalai Tesque Inc.), ion‐exchanged water (H_2_O) as a solvent, hexamethyldisiloxane (HMDS, (O(Si(CH_3_)_3_)_2_)) and acetic anhydride (AA).

### Preparation of β‐Zeolite and β‐Zeolite‐Containing Hierarchical Catalysts Using Gel Skeletal Reinforcement Method

According to the flowchart shown in Figure S4, β‐zeolite was prepared. NaAlO_2_ of 0.817 g, H_2_O of 0.585 g, TEAOH of 18.97 g and NaOH of 0.583 g were added in a 200 mL beaker. The mixture was stirred at room temperature, 31.70 g of colloidal silica was added dropwise to the mixed solution with stirring at room temperature for 30 min, and then the resultant mixture was transferred to a stainless‐steel sealed container and was crystallized under hydrothermal conditions at 142 °C for 20 h. Subsequently, centrifugation was performed at 3000 rpm for 10 min to remove the supernatant, and the precipitate was washed with ion‐exchanged water until the pH was 7 to 8. After washing, the C‐Beta (C: Conventional) was dried at 77 °C for overnight and finally calcined under the conditions of dry air flow rate 600 cm^3^/min, heating rate 2.29 °C/min, temperature 550 °C and retention time 5 h.

β‐zeolite‐containing hierarchical catalysts using the gel skeletal reinforcement (GSR) method was prepared according to the flowchart shown in Figure S5. The fabrication of GSR‐3.2HS−C‐β is explained as an example. First, a mixture of 0.817 g of NaAlO_2_, 0.585 g of H_2_O, 18.97 g of TEAOH, and 0.583 g of NaOH is stirred in a 200 mL beaker at room temperature, and colloidal silica of 31.70 g was added dropwise in the mixed solution, and the mixture was stirred at room temperature for 30 min. Thereafter, the precursor solution was placed in a PFA container containing a mixture of HMDS of 0.8315 g and AA of 1.3225 g, and aging was performed at 50 °C for 48 h. Subsequently, it was transferred to a stainless‐steel sealed container and was crystallized under the hydrothermal condition at 142 °C for 20 h. Subsequently, centrifugation was performed at 3000 rpm for 10 min to remove the supernatant, and then the precipitate was washed three times with ion‐exchanged water. After washing, the GSR‐3.2HS−C‐β (C: Conventional) was prepared by drying at 77 °C for 1 day and finally calcining at 550 °C for 5 h.

GSR‐3.2HS−C‐β is the sample name, in which GSR represents gel skeletal reinforcement, 3.2 represents the ratio of HMDS x100/SiO_2_ in colloidal silica (mol/mol), HS is the abbreviation of HMDS, C means conventional and β is the type of zeolite. GSR‐2.6HS−C‐β and GSR‐2.9HS−C‐β were prepared similarly with changing the amounts of HMDS and AA added.

Since the cation type of GSR‐3.2HS−C‐β was Na^+^, the cation exchange was performed to the H^+^ type via the NH_4_^+^ type for catalysis use according to the flowchart shown in Figure S5 using ammonium nitrate (NH_4_NO_3_; manufactured by Nacalei Tesque Inc.) as a reagent and ion‐exchanged water (H_2_O) as a solvent. Ammonium nitrate (2.40 g) was dissolved in ion‐exchanged water (30.00 g) and Na^+^‐type of the catalyst (3.00 g) was added to it and was stirred at 80 °C for 2 h. Thereafter, washing was performed using ion‐exchanged water until the pH of the filtrate was 7 to 8 by suction filtration. The washed sample was dried at 77 °C for 1 h. After repeating the above step three times, the sample solid was calcined at 550 °C for 4 h. The sample after ion exchange was denoted as GSR‐3.2HS−H‐β. GSR‐2.6HS−H‐β and GSR‐2.9HS−H‐β were prepared similarly.

### Characterization of β‐Zeolite and β‐Zeolite‐Containing Hierarchical Catalysts Using Gel Skeletal Reinforcement

In order to investigate the crystalline state of the produced catalyst powder, X‐ray diffraction (XRD) measurement was performed. Ultima IV (RIGAKU Electric Co., Ltd.) was used as the X‐ray diffractometer, and a sample of 0.10 g in a powder state was fixed to a holder using a glass slide. As the X‐ray source, CuKα rays (λ=0.15405 nm) monochromatic with a Ni filter were used, and the measurement was performed at 40 kV–20 mA, sampling width 0.02, slit 2/3°, scattering slits 2/3°, light receiving slit 0.45 nm, can speed 4°/mins, and measurement range 2θ=3–70°.

X‐ray fluorescence analysis was performed to investigate the silica‐alumina ratio of each sample. The X‐ray fluorescence spectrometer, ZSX Primus II (Rigaku Co., Ltd.) was used. The determination of SiO_2_, Al_2_O_3_ and Na_2_O was performed using catalyst powder and PP film under vacuum of <20Pa.

N_2_ adsorption and desorption measurements were performed to examine BET surface area, t‐plot micropore surface area, t‐plot external surface area t‐plot micropore volume, total pore volume, average pore diameter, BJH surface area, BJH pore volume, BJH pore diameter of each sample. First, the sample of 0.080 g was set in the sample tube, set to 350 °C, and pretreated under 3 h vacuum exhaust. Thereafter, the adsorption and desorption isotherms of nitrogen gas at 77 K was measured using BELSORP‐mini II (Japan Bell Co., Ltd.).

In order to observe the structure of the prepared catalyst, observation by TEM was performed. A transmission electron microscope JEM‐1011 (manufactured by Japan Electronics Co., Ltd.) was used for the photographs. The shooting time is 30 s.

NH_3_‐TPD (temperature‐programed desorption) was measured to evaluate the amount of acid sites of catalysts used. First, a sample of 40 mg was packed into a fixed‐bed reactor, was heated to 600 °C at 10 °C/min under He stream of 30 cc/min, and was held for 3 h. Then, the reactor was cooled to 100 °C, and pulses of NH_3_ were introduced using a 1.0 cm^3^ loop into the catalyst layer until an area of one NH_3_ pulse became same. After the catalyst was held at 100 °C under the He stream for a while, the temperature was raised to 650 °C at a heating rate of 10 °C/min to measure NH_3_‐TPD using a gas chromatography with thermal conductivity detector (GC‐TCD, Shimadzu GC‐8 A). The measurement conditions are as follows: INJ/IT 170 °C, COL 140 °C, ATTN 16, Current100 mA, Column flow 30 cc/min, Column filler porapack Q 1 m.

The amount of coke deposited on the catalyst after the reaction was measured using TG‐DTA (Thermogravimetry‐differential thermal analysis, DTG‐60AH, Shimadzu) under the conditions: Sample weight 10 mg, heating rate 10 °C, measuring range 25–600 °C, a pan Al, a flow rate of dried air 100 ml/min.

### Catalytic Cracking of *n*‐Dodecane by β‐Zeolite and β‐Zeolite‐Containing Hierarchical Catalysts Using Gel Skeletal Reinforcement

Catalytic cracking of *n*‐dodecane was performed using a conventional fixed bed flow reactor as shown in Figure S6. 1.0 g of the calcined sample was sandwiched between quartz wool and quartz sand, packed in the reactor, and the temperature was raised to 500 °C at 5 °C/min while nitrogen was introduced into the reactor at a flow rate of about 30 cc/min. Next, as a model compound, *n*‐dodecane (CH_3_(CH_2_)_10_CH_3_, Nacalai TesqueInc.) was introduced into the reactor at about 1.3 ml/min for 80 s, and nitrogen was again flowed into the reactor for 30 min at about 30 cc/min. Gaseous products were recovered with nitrogen in a gas bag, and liquid products were collected in a cold‐trap tube dipped in ethanol at 0 °C for the first 15 min and water at 15 °C for the next 15 min. After completion of the reaction, the catalyst packed in the reactor was recovered for the measurement of the coke weight by TG‐DTA measurement described above.

The gaseous and liquid products were analyzed using a gas‐chromatography‐flame ionization detector (GC‐FID, Shimadzu GC‐2014 for gaseous products and Shimadzu GC‐2010 for liquid products). The measurement conditions are shown below: Injection temp. 250 °C, injection mode split, carrier He, pressure 100.6 kPa, full flow rate 170.9 mL/min, column flow rate 0.83 mL/min, linear velocity 16.8 cm/sec, purge flow rate 4.0 mL/min, split ratio 200.0, column temp. 0.0 °C, equilibrium time 3.0 min, detector temp. 320 °C, columns used BP‐1, column length 60.0 m, column inner diameter 0.25 mm, thickness of the liquid phase 0.50 μm, column initial temp. 0 °C, 16 min, heating rate 2 °C/min (114 min), final temp. 228 °C.

Prior to product analysis, the analysis of a standard gas (GL Science Co., Ltd., methane 1.01 %, ethane 1.02 %, propane 1.01 %, *i*‐butane 1.02 %, *n*‐butane 1.01 %, carbon dioxide 1.01 %) was performed under the same condition. Using a gaseous sample of 30 *μL* was introduced into the GC with a 100 *μL* syringe, and for the measurement of liquid products, 0.2 *μL* was used in an autosampler (AOC‐20i; Shimadzu Corporation). The data obtained by GC analysis were used to identify peaks from the retention time.

The conversion (%) of the catalytic cracking reaction was calculated by the total weight of the recovered products (g) ÷ the *amount of n‐dodecane introduced (*g) ×100.

After catalytic cracking of *n*‐dodecane, thermogravimetric analysis was performed in order to investigate the amount of coke generated on the used catalyst. The amount of coke was calculated by the weight change of 400–600 °C due to the combustion of coke.

## Conflict of Interests

The authors declare no conflict of interest.

1

## Supporting information

As a service to our authors and readers, this journal provides supporting information supplied by the authors. Such materials are peer reviewed and may be re‐organized for online delivery, but are not copy‐edited or typeset. Technical support issues arising from supporting information (other than missing files) should be addressed to the authors.

Supporting Information

## Data Availability

The data that support the findings of this study are available in the supplementary material of this article.
