# Supplementary material for: Preparation of Novel Hierarchical Catalysts by Simultaneous Generation of β‐Zeolite and Mesoporous Silica for Catalytic Cracking
Source: Chempluschem. 2024 Oct 29;89(12):e202400447. doi: 10.1002/cplu.202400447 (PMC11639649; doi:10.1002/cplu.202400447)
Supplement: Supplementary file 1 — Supporting Information [file CPLU-89-e202400447-s001.pdf]

ChemPlusChem

Supporting Information

Preparation of Novel Hierarchical Catalysts by Simultaneous Generation of β-Zeolite and Mesoporous Silica for Catalytic Cracking

Haruna Oshimura, Shuuma Tanaka, Shouya Nagata, Shinya Matsuura, Tadanori Hashimoto, and Atsushi Ishihara*

Table S1 Pore properties of each sample obtained by nitrogen adsorption-desorption measurement

Catalyst	BET Surface Area (m^2/g)	t-plot micropore surface area (m^2/g)	t-plot external surface area (m^2/g)	t-plot micropore volume (cm^3/g)	Total Pore volume (cm^3/g)	Average Pore diameter (nm)	BJH Surface area (m^2/g)	BJH Pore volume (cm^3/g)	BJH Pore diameter (nm)
C-β	661	629	38.7	0.27	0.50	3.02	38.6	0.23	3.3
GSR-2.6HS-C-β	658	629	20.3	0.26	0.41	2.50	20.4	0.12	3.7
GSR-2.9HS-C-β	571	516	51.6	0.22	0.58	4.08	50.7	0.36	3.3 (18)
GSR-3.2HS-C-β	375	295	83.7	0.13	0.94	9.99	81.1	0.81	21 (52)
GSR-3.5HS-C-β	292	155	136	0.07	1.25	17.1	136	1.18	38
GSR-3.8HS-C-β	254	157	99.9	0.07	1.15	18.0	99.8	1.07	52 (33)

Prior to the experiment, 0.040g of a sample was heated at 350°C in vacuum for 3 h in Belprep II (BEL Japan, Inc.). Then, adsorption and desorption isotherms were obtained at 77K using Belsorp Mini II (BEL Japan, Inc.). Average pore diameter was calculated from values of BET surface area and total pore volume.

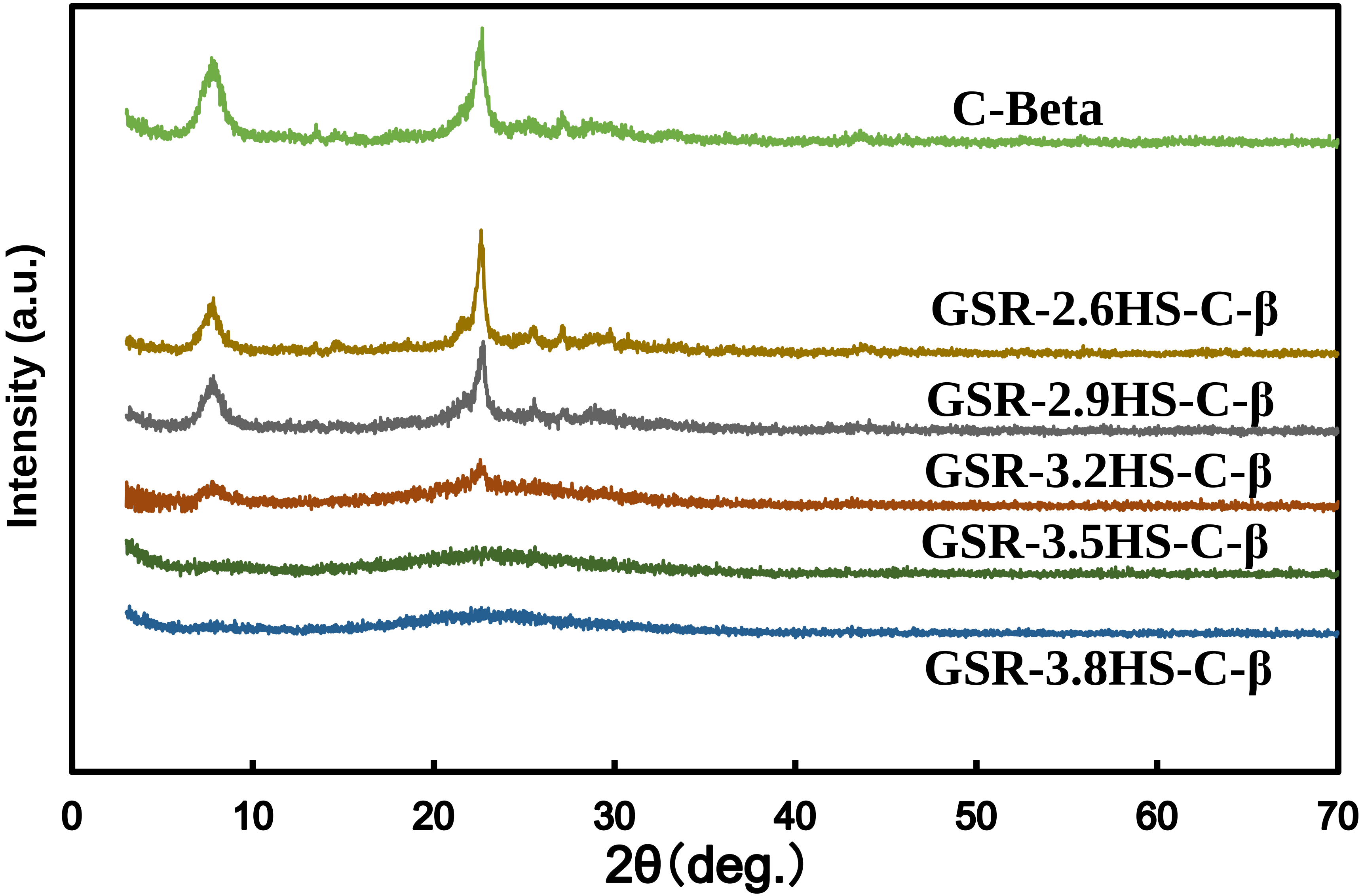

Fig. S1 XRD patterns of H-β-zeolite containing hierarchical catalysts

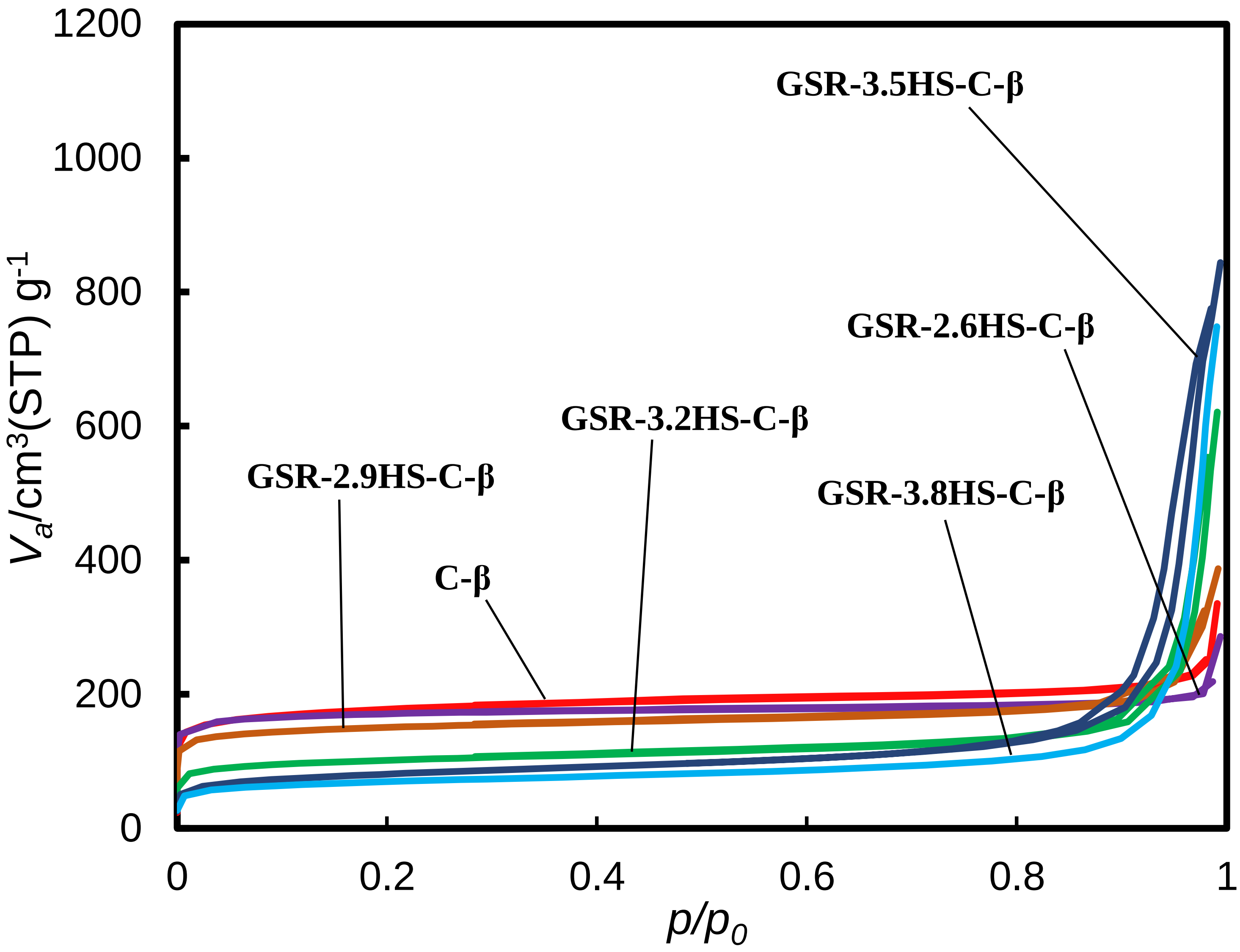

Fig. S2 Nitrogen adsorption-desorption isotherms.

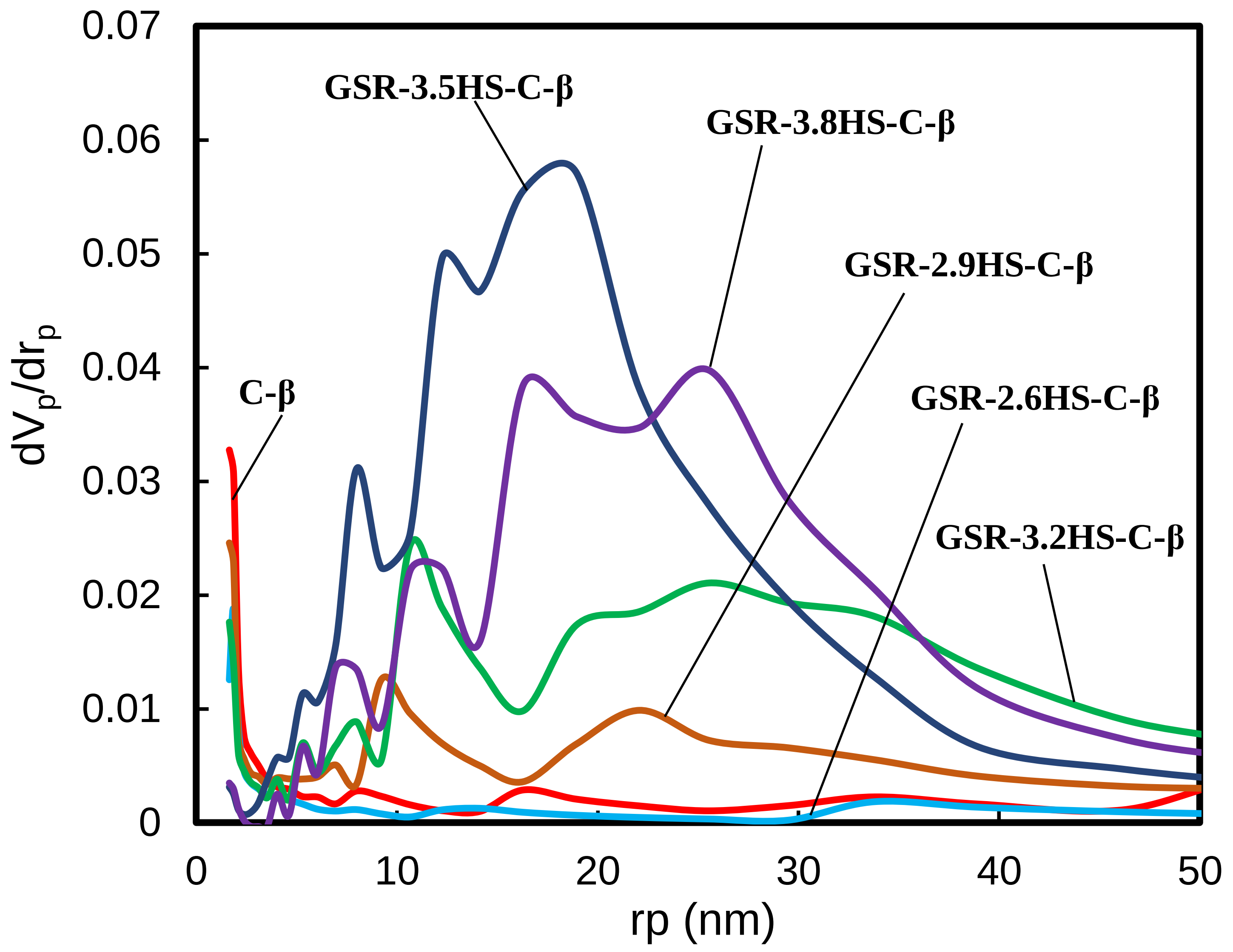

Fig. S3 BJH pore-size distribution.

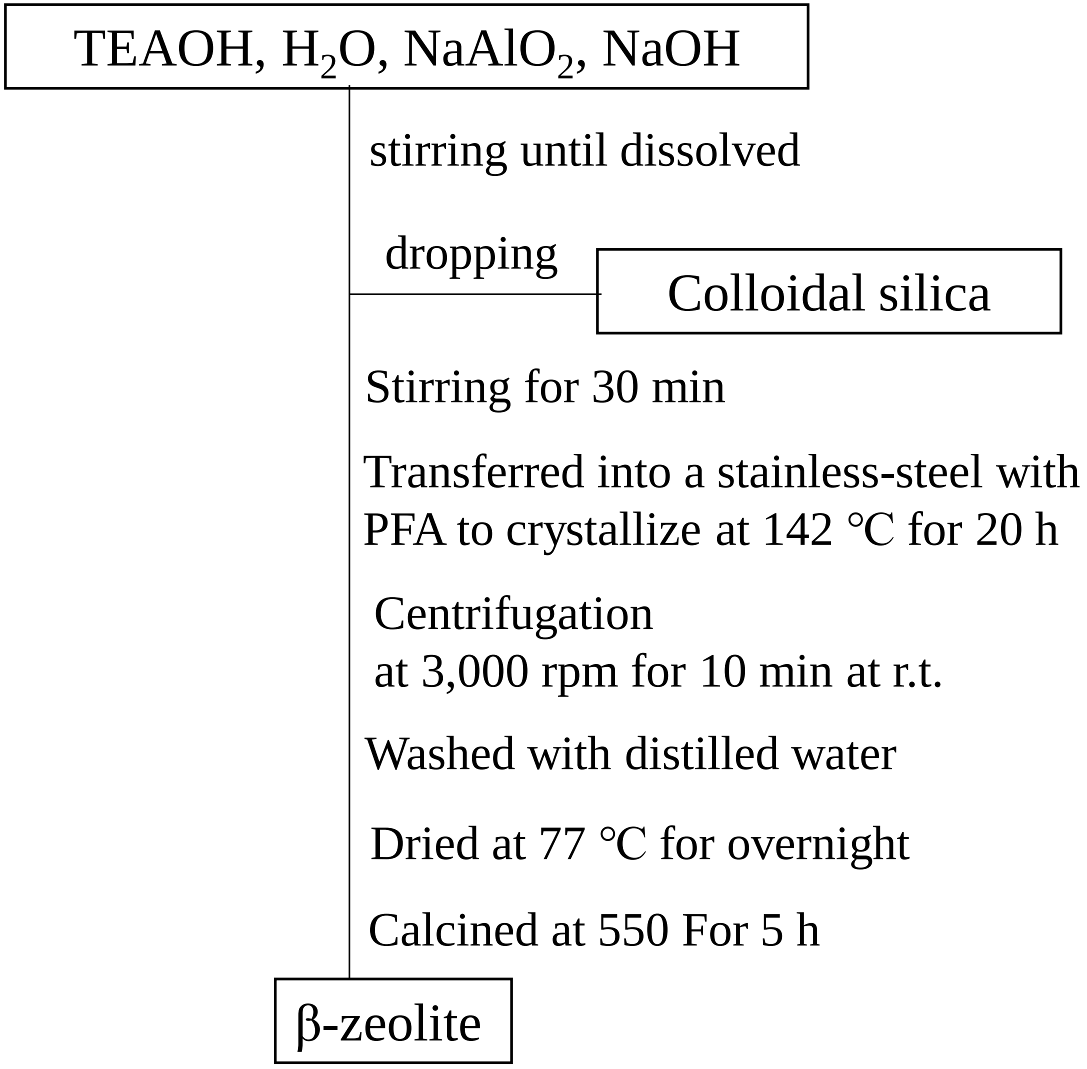

Fig. S4 Flowchart for preparation of Beta

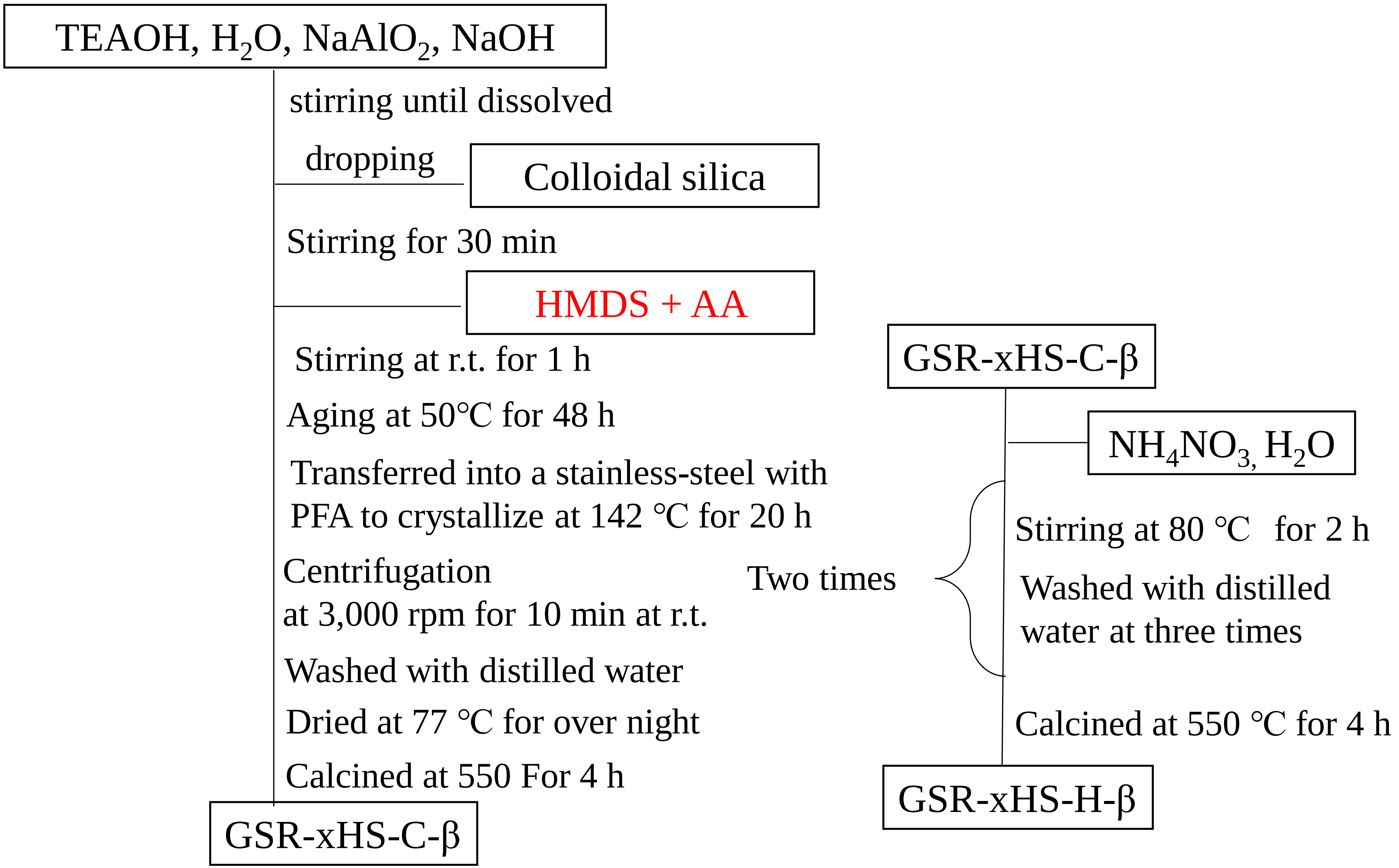

Fig. S5 Flowchart for preparation of Beta
HS: HMDS; x=2.6, 2.9 or 3.2, the ratio of HMDS x 100 / SiO_2 in colloidal silica (mol/mol).

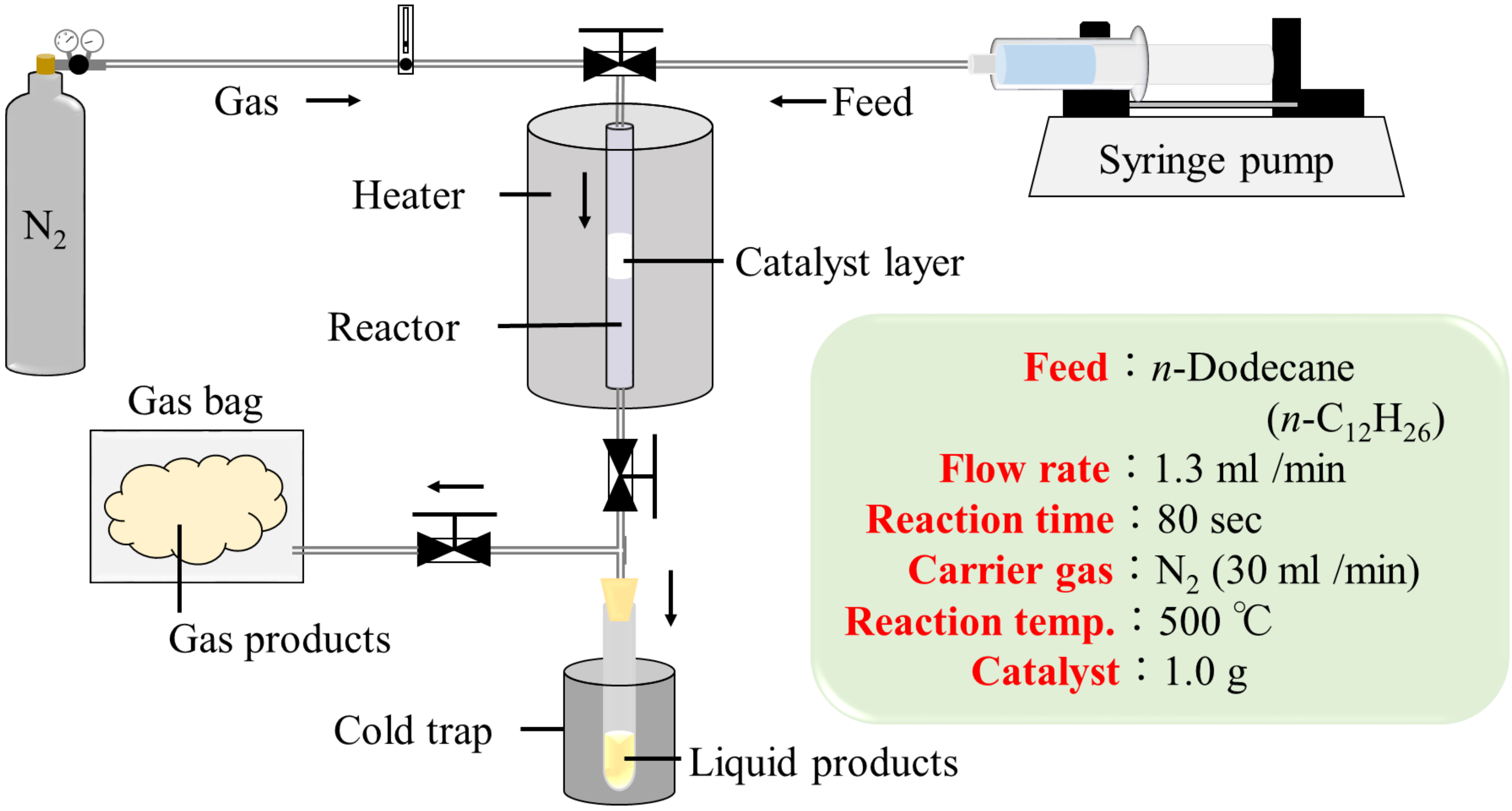

Fig. S6 Reaction apparatus of catalytic cracking.
